# The unconventional regulation of non-muscle myosins

**DOI:** 10.1007/s12551-026-01437-8

**Published:** 2026-05-08

**Authors:** Andrew T. Lombardo

**Affiliations:** Jacobs School of Medicine and Biomedical Sciences, Department of Biochemistry, University at Buffalo, Buffalo, NY USA

**Keywords:** Molecular motors, Mechanobiology, Myosins, Actin, Cytoskeleton, Cell polarity

## Abstract

Myosins are actin-based molecular motors that power diverse forms of cellular motility across life. Initially characterized as the contractile machinery of muscle, the superfamily now includes numerous non-muscle classes with distinct cellular functions. Over the past decades, work across numerous actin-based systems has uncovered multiple modes of non-muscle myosin control. Recent advances now expand and, in some cases, challenge conventional regulatory paradigms, underscoring the complexity and adaptability of myosin function in cellular contexts. In this short review, I begin with a concise summary of the major canonical regulatory systems for non-muscle myosins. I follow this by highlighting the major novel regulatory findings from the last decade specifically relating to protein-binding partner activation, structural lipid-binding motifs, co-assembly of mixed classes of myosins, and the regulation of multi-motor complexes by the nanoscale organization of cellular actin. This review will appeal to the general scientific reader aiming to understand the mechanisms regulating myosins outside of the context of muscle and the current state of the non-muscle myosin field.

The myosin motor domain is a versatile molecular motor which converts ATP hydrolysis into mechanical force to generate movement along actin filaments. This domain is incorporated into numerous essential gene products in plants, fungi, and animals. A great deal of the motion observed in higher order life can be ascribed to the interactions between myosin and actin with ATP. The locomotion of amoeba, cytoplasmic streaming of plants, polarized secretion of yeast, and muscle contraction all depend on the intricate regulation of this interaction. For at least the first 104 years of study (Kühne 1864) myosins were identified as the contractile component of muscle responsible for force, tension, and motion generation (Adelman and Taylor 1969; Hatano and Tazawa 1968). In muscle, thick bundles of myosin slide past filamentous actin (also called microfilaments or F-actin) within a highly ordered myofibril, the contractile organelle of muscle. The highly ordered structure of muscle is essential for regulating interactions between myosin and actin, which is physically prevented by tropomyosin in a calcium-sensitive system. In early studies, other animators of life, such as the bacterial flagellar rotation and the ciliary beating were thought to occur through muscle-independent mechanisms (Astbury and Weibull 1949). While the molecular basis of other forms of biological motility was unknown, some proposed that actin-myosin-like systems outside of muscle could exist (Hatano and Oosawa 1966; Loewy 1952). A watershed moment occurred in the late 1960s with the characterization of contractile myosins from cells devoid of myofibrils (Adelman and Taylor 1969; Hatano and Tazawa 1968; Pollard and Korn 1973).

Upon the realization that all eukaryotes had multiple myosin proteins serving differing purposes, a formal nomenclature was developed to distinguish between myosin classes (Pollard et al. 1974). A more casual distinction was made which persists to this day: “muscle myosins” refer exclusively to class 2 myosins found in skeletal, cardiac, and smooth muscle, while “non-muscle myosins” are any other myosins regardless of their localization or tissue expression (including in muscle cells). Yet non-muscle myosin regulation remained elusive, where the state of the field was summarized by (Pollard, et al. 1974): “[…] one cannot be certain of even the *general* aspects of the control mechanism in any non-muscle cell.” (Pollard et al. 1974). Over the following decades, a major scientific endeavor was undertaken to understand how non-muscle myosins could be regulated without the structural order of the muscle myofibril. By 1983, the intestinal brush border, and its epithelial microvilli, had been identified as the most highly ordered actomyosin model system outside of muscle (Bretscher 1983). Through studies of microvilli, and later the multitude of additional actin-based non-muscle cellular structures, numerous other non-muscle myosins were identified and characterized. Decades of study defined the general principles that control the regulation of both muscle and non-muscle myosins (Tyska and Warshaw 2002). More recently, new insights into the regulation of non-muscle myosins have advanced rapidly (see the following comprehensive review of the field 10 years prior to this review (Heissler and Sellers 2016). These recent advances expand upon, and challenge, the conventional regulatory mechanisms and are the focus of this review.

## Established regulatory mechanisms of myosins

**Fig. 1:**
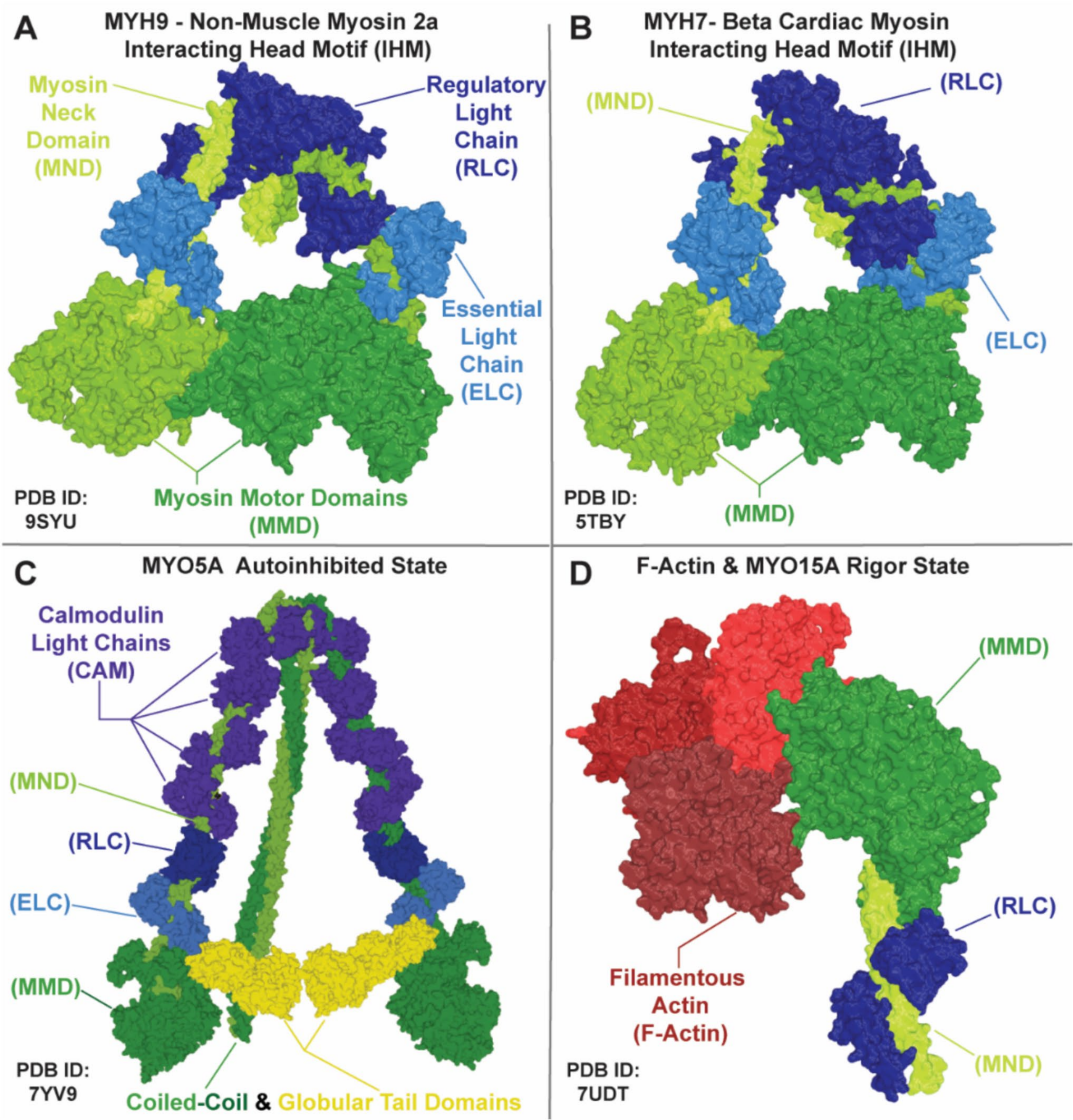
Myosin surface structures in differing regulatory states. **A** Surface structure of NM-Myo2A in the inactive state adapted from PDB ID: 9SYU (PDB DOI: https://doi.org/10.2210/pdb9syu/pdb) (Casas-Mao et al. 2026). NM-Myo2A forms a hetero-hexamer comprised of two myosin MHY9 motors (greens), where two calmodulin-like essential light chains (ELC: blue), and two calmodulin-like regulatory light chains (RLC: purple) bind to the myosin neck domains (MND: lime-green). In the absence of light chain phosphorylation, the motors form a folded inactive state involving the direct molecular binding between the myosin motor domains termed the Interacting Head Motif (IHM)**.** The IHM myosin motor domain positioning in NM-Myo2A is remarkably similar to the IHM state of Beta Cardiac Myosin shown in **B** adapted from PDB ID: 5TBY (Alamo et al. 2017). Instead, the predominant structural changes between the IHM in MHY9 and MHY7 involve the positioning of the myosin tail domain (Casas-Mao et al. 2026) (tails shortened for clarity, however, note the angle change of the coiled-coiled (CC) tail following the RLC binding site) potentially due to the regulatory effect of myosin binding protein-C in cardiac muscle (Nelson et al. 2020, 2023). Many non-muscle myosins, including myosin 5a **C** are predicted to form an autoinhibited state where regulatory regions of the tail (yellow) interact with the myosin motor domain (adapted from PDB ID: 7YV9) (Niu et al. 2022). Both the IHM and autoinhibition are relieved through a combination of post-translational modifications (e.g., phosphorylation) and molecular interactions between lipids or protein binding partners specific to each myosin. Once activated, non-muscle myosins are free to bind to actin filaments (F-actin: reds) in an ATP-dependent manner as shown in **D** of Myosin 15a adapted from PDB ID: 7UDT (Gong et al. 2022). Note that the genetic identity of the first light chain following the motor domain in Myo15a was reported to be an RLC providing an uncharacterized, potential regulatory mechanism for the motor (Gong et al. 2022). Structural data were obtained from the Research Collaboratory for Structural Bioinformatics Protein Data Bank (RCSB PDB) and produced using Version 2.3.0 Open-Source PyMOL Molecular Graphics System, Version 3.0 Schrödinger, LLC (Schrodinger 2015)

To understand recent advances in non-muscle myosin regulation, one must first understand the well-established structural and biochemical properties of myosins. This short review covers the broad essentials of established properties and regulatory mechanisms of non-muscle myosins with citations for further in-depth study. Humans have 40 myosin genes, which generate further functional diversity through their differential expression, alternative splicing, and post-translational modifications. Of these, 38 produce established protein gene products, while two are suspected pseudogenes: The MYH16 pseudogene product encodes a motorless truncation of unknown function reportedly expressed only in human jaw muscles (Toniolo et al. 2008). The other pseudogene, Myo15B, has been detected at the transcript level; however, the protein product status is currently uncertain (Boger et al. 2001). Homologues of the genes are found in plentiful abundance, across the tree of life, and are grouped into classes of myosins based on shared molecular structure and peptide sequence identity (Odronitz and Kollmar 2007). Each class shares a structurally defined myosin motor domain almost exclusively located toward the N-terminus of the protein, but otherwise varies widely in its structure, expression, molecular function, regulation, and cellular localization (Fig. 1) (Chinthalapudi and Heissler 2024; Geeves 2016; Houdusse and Sweeney 2016; Quintanilla et al. 2023).

Each myosin can be described structurally in three basic components, motor, neck, and tail (Fig. 1A). Some myosins are described as having a fourth rod domain region located between the neck and tail required for dimerization, such as the coiled coil of myosin Va (Fig. 1C) (Trybus 2008). These components are subject to multiple regulatory processes, which are specific to each myosin. Nonetheless, all myosins share key regulatory principles: (1) They bind calmodulin-like light chains (Heissler and Sellers 2014). (2) They exhibit modified enzymatic and structural behavior upon binding actin (Geeves 2016). (3) They can form an autoinhibited structure (Liu et al. 2006), (4) Their ATPase activity is sensitive to applied forces, meaning they are force transducers (Tyska and Warshaw 2002). Thus, in its simplest form, myosins are multi-allosteric enzymes (Heissler and Sellers 2016). These core regulatory principles are capable of simultaneous cooperative or antagonistic effects. Many of the “novel” regulatory mechanisms discussed in this review arise from combinations of these four key regulatory components, often in emergent or sometimes unexpected ways. Additionally, new mechanisms specific to each myosin class have been recently described. The complexity of the cellular environment, and the reality that study on Earth involves the application of gravitational force (to which myosins detect and respond (Harrison et al. 2003)) has made the true isolation of all regulatory mechanisms nearly impossible. However, clever investigation has brought great insight into the individual roles of these shared regulatory systems in myosins.

### Regulation through calmodulin-like light chains

All myosins bind at least one light chain via their neck or lever-arm region, using IQ motifs with the consensus sequence IQxxxRGxxxR. Different classes of myosin contain different numbers of light chain-binding IQ motifs depending on their cellular function. For example, class 2 myosins contain two IQ motifs while myosin Va, a cargo transporter, contains six. Each motif binds calmodulin (CaM) or a calmodulin-like light chain which has lost the ability to bind calcium (Heissler and Sellers 2014; Odronitz and Kollmar 2007). The genetic identity of these light chains can vary with humans having at least 13 currently characterized myosin light chain genes (which follow the nomenclature MYL1, MYL2, etc.), three human CaM genes (CALM1, CALM2, CALM3), and the characterization of additional calmodulin-like gene products also competing for myosin heavy chain-binding domains (Heissler and Sellers 2014). Additionally, a single myosin may bind multiple different light chains derived from distinct genes (Coluccio and Bretscher 1990). The binding of the light chains provides structural stabilization and mechanical rigidity to the neck domain (Tyska and Warshaw 2002). This rigidity functions to amplify small conformational changes in the motor domain to generate large working strokes needed to effectively displace actin. Furthermore, the binding, phosphorylation, regulation by divalent cations, and genetic identity of the light chains are a major category of myosin regulation, and in-depth exploration of this topic can be found in prior reviews (Heissler and Sellers 2016).

### Actin-associated regulation and actin-activated ATPase activity

The enzymatic ATPase cycle of myosin is highly conserved. However, the biochemical rates and equilibrium constants vary widely between different classes (De La Cruz et al. 2001; Dosé et al. 2007; Ostap and Pollard 1996). Systematic investigation through biophysical and biochemical approaches has characterized the rates of each step of ATP hydrolysis for several human myosins (De La Cruz and Ostap 2009). Despite these variances, all myosins exist in multiple enzymatic or regulatory states historically termed “relaxed” (i.e., not able to bind to actin) or “active” (i.e., able to bind to actin), originating from muscle studies. At the core of this multi-state model is the conceptual framework that all myosins complete their ATPase cycle more rapidly when bound (or able to bind) to F-actin compared to when F-actin is not available. This actin-activated ATPase activity is an adaptation that ensures myosin activity is turned on only when engaged productively with its track.

Through decades of study, the laboratory of Roger Cooke championed the existence of a third state termed the super-relaxed state (SRX) (Cooke 2011; McNamara et al. 2015). This state was first identified in rabbit skeletal muscle through the identification that the heat produced by resting muscle approached 5 × less than the expected energy expenditure of the myosin relaxed ATPase rate (Ferenczi et al. 1978). The characterization and wide acceptance by the cytoskeletal motor field of the SRX state has come relatively recently (Stewart et al. 2010) and had massive implications on our understanding of both muscle and non-muscle myosin regulation (McNamara et al. 2015). For further information on actin-activated ATPase states and myosin energy expenditure, several excellent reviews have been produced elsewhere (Alamo et al. 2018; Cooke 2011; Trivedi et al. 2018). In muscle myosins and several classes of non-muscle myosins, an analogous low-ATPase-usage state involves a structurally folded-back state termed the interacting-heads motif (IHM) (Fig. 1A, B) (Craig et al. 1983; Trybus et al. 1982; Wendt et al. 2001). The advances that have arisen from the characterization of the SRX state are a current area of intense investigation. Specifically, the relationship between the SRX and IHM states remains unclear currently (Nag and Trivedi 2021). Other mechanisms of actin-associated regulation include actin isoform specificity (Müller et al. 2013), myosin sensitivity to the bound nucleotide state within the actin monomers (Zimmermann et al. 2015), and sensitivity to actin-bound tropomyosin (Barua et al. 2014; Pruyne et al. 1998).

### Force sensitivity

All myosins are force transducers capable of detecting applied forces and responding with a measurable biochemical response. The transduction, in its simplest form, involves the changing of the motor domain’s ATPase rate in response to a force applied parallel to the axis of the actin-bound filament (Rock et al. 2001; Tyska and Warshaw 2002). The force transduction property of myosin is independent of the isoform and genetic identity of the actin filament (Harris and Warshaw 1993). Instead, “assistive loads” (applied forces in the same direction as the motor’s motility) accelerate the release of ADP within the myosin motor domain while “resistive loads” (forces applied in the direction opposite the motility of the motor) result in the slowing of the rate of ADP release (Hooijman et al. 2011; Veigel et al. 2003). ATP binding and hydrolysis are not independent of load, but are altered to a much lower extent in response to applied force on the myosin motor domain compared to ADP release (Kad et al. 2007; Veigel et al. 2003).

## Recent advances in novel mechanisms for non-muscle myosins

### Structural lipid-localization motifs

**Fig. 2:**
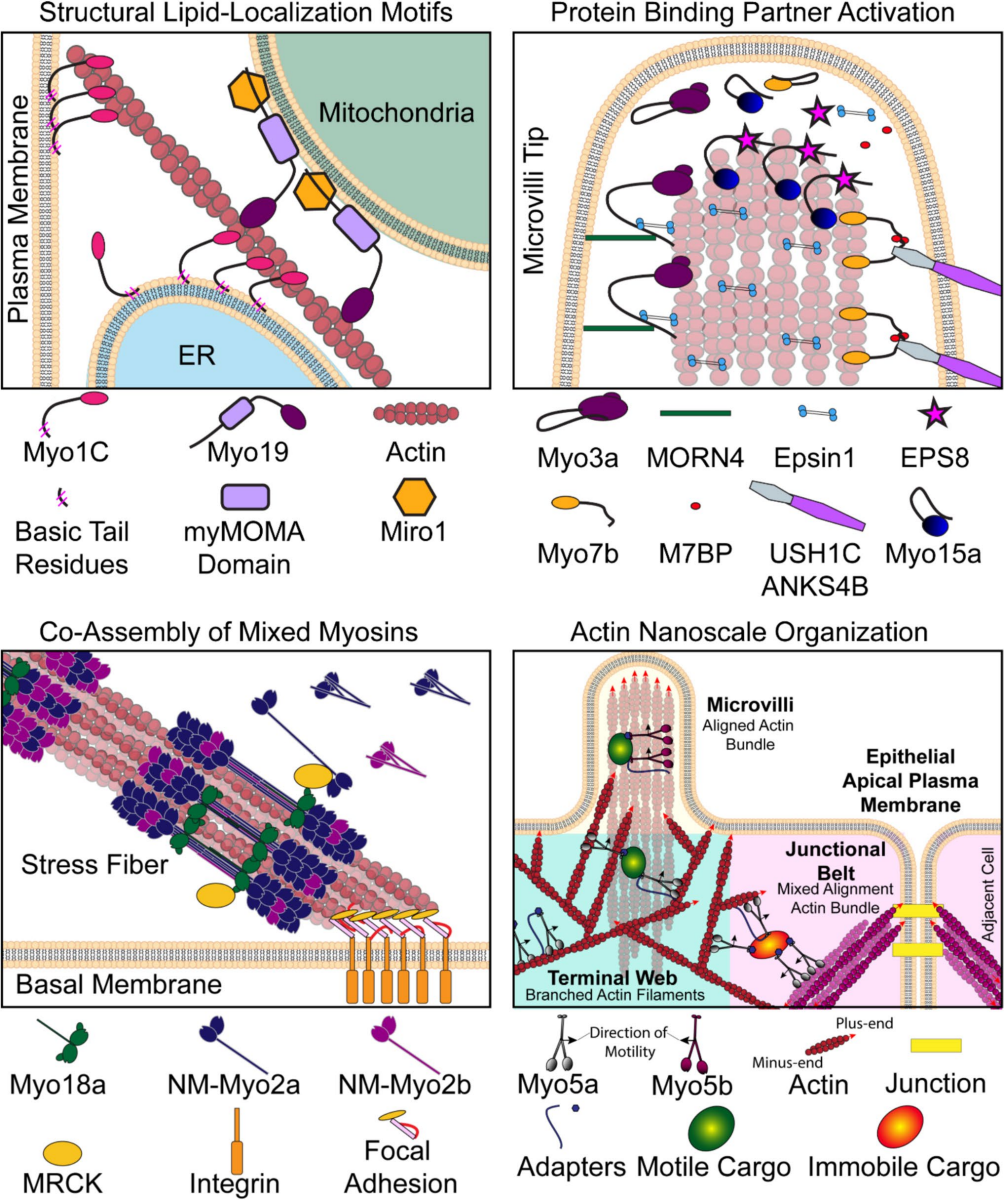
Selected Myosin Regulatory Mechanisms. Schematic representations of the myosin regulatory mechanisms discussed in this review are shown. Recent advances highlighted include: (i) characterization of lipid-targeting residues in class I myosins and of the MyMOMA domain in Myo19; (ii) identification of activating binding partners for class III, VII, and XV myosins; (iii) discovery and characterization of mixed contractile bundles containing both class II and class XVIII myosins; and (iv) delineation of the emergent mechanical and trafficking properties exhibited by teams of Myo5A and Myo5B/C motors navigating complex three-dimensional actin networks

The myosin tail domain contains the highest degree of structural and sequence variance across different classes. This variance allows for the specialization of each motor type, especially through the incorporation of targeting modules and specific receptor binding sites. Possibly the most established among these are the membrane-binding domains found at the C-terminal region of the myosin-1 gene products (McIntosh and Ostap 2016). This domain confers direct binding to phosphoinositides incorporated into lipid membranes for essentially all myosin-1 proteins (Komaba and Coluccio 2010). New results have identified novel targeting mechanisms in myosin-1 and have expanded the list of myosins with similar targeting mechanisms. Specifically, the targeting of myosin-19 to mitochondria was characterized to occur through a myMOMA domain into the tail, where the overall basic charge of an 83-amino acid region is required for localization (Hawthorne et al. 2016). Surprisingly, mutating just two residues (R882 and K883 in humans) can redirect the protein to the endoplasmic reticulum (Fig. 2). The mechanism by which these residues could selectively target Myo19 to mitochondria vs. the endoplasmic reticulum is unclear. Nevertheless, this finding raises the possibility that individual basic residues can confer organelle specificity, rather than simply mediating general membrane association.

Applying this principle may help explain the distinct cellular localizations of the eight human myosin‑1 genes. For example, Myo1E contains a basic-residue region which is responsible for localization to lamellipodial tips (Tanimura et al. 2016). Alternatively, Myo1C is targeted to sites of cell-cell contact through a critical pair of basic residues (K892 or R903) that enable direct phosphatidylinositol-4,5-bisphosphate (PI(4,5)P_2_) binding (Kannan and Tang 2018) (Fig. 2). One potential reason for maintaining eight distinct myosin-1 genes is that each has unique mechanical properties when bound to actin (Greenberg et al. 2015). By targeting specific myosin-1 gene products to different cellular membranes, the cell could potentially tune the mechanical properties applied to each structure. This potential mechanism remains to be fully established.

How can individual residues within a larger basic residue region direct lipid-binding myosins to specific membrane locations or distinct actin networks? A current hypothesis involves the coordination of the molecular interactions between the myosin and scaffolding proteins. Some recent evidence supports this mechanism (Arden et al. 2025; Boguslavsky et al. 2012; Petzoldt et al. 2012; Sun et al. 2021), which is analogous to the common myosin theme of binding adaptor proteins through myosin-tail-mediated protein-protein interactions (Akhmanova and Hammer 2010). This system can include the activation of the motor upon binding to the adaptor (Sckolnick et al. 2013) (further discussed below).

However, another hypothesis is that myosin targeting is regulated through the binding of specific lipids or phosphoinositides independent of adaptor-protein or scaffolding-protein recruitment.

Among lipid-associated myosins, five human myosin genes contain MyTH4-FERM (myosin tail homology 4-band 4.1, Ezrin, Radixin, Moesin) domains (Myo7a, Myo7b, Myo15a, Myo15b, and Myo10). However, the existence of the Myo15b pseudogene product is not clear, leaving only four confirmed MyTH4-FERM-expressed proteins in humans (Weck et al. 2017). The FERM (4.1 Ezrin, Radixin, Moesin) domains of these proteins are conserved within each myosin class across wide swaths of the tree of life, yet they retain key structural differences between classes (Planelles-Herrero et al. 2016). An in-depth structural analysis of the MyTH4-FERM domains of myosins across multiple species concluded that class-specific insertions to the MyTH4-FERM modify surface features and shift the relative positioning of subdomains, creating novel interaction sites for binding partners and specific lipid membrane components (Planelles-Herrero et al. 2016). Amazingly, a recent study identifies that a specific lipid-binding domain is not essential for all myosin-lipid associations (Montanarella et al. 2025). Instead, nearly the entire tail of Myo6 contributed to picomolar binding of the lipid cardiolipin. The finding that a multi-domain, ~ 400 amino acid region of human Myo6 (aa 835–1253) was sufficient and necessary to bind cardiolipin indicates that there remains much more to learn about myosin-lipid regulatory interactions. Nonetheless, the current consensus is that these mechanisms are not mutually exclusive but instead combine to create a complex assortment of targeting and activation systems.

### Protein binding partner activation

Regulation of myosins by accessory protein binding is well established. Examples of class and function-specific binding of adaptor proteins to the tail of myosin are numerous (Krendel and Mooseker 2005). Canonical examples include the association of Rab-27a with myosin-5a coordinated through melanophillin, which results in a structural unfolding of the motor, attachment to pigment granules, biochemical activation of the motor, and enhanced run lengths through tethering of the motor to the actin (Liu et al. 2006; Sckolnick et al. 2013; Thirumurugan et al. 2006; Van Gele et al. 2009). New research on Myo6 supports a similar mechanism of activation for the minus-end-directed motor (Shang et al. 2017; Tomatis et al. 2017). Activation can also involve the binding of oligonucleotides as is the case in the yeast motor Myo4p which requires messenger RNA to form a motile complex (Sladewski et al. 2013; Sladewski and Trybus 2014). In addition to class-specific binding partners are some assumed interactors. Calmodulin-like light chains are largely referred to as part of the myosin in most manuscripts; however, they are distinct gene products which can be exchanged (Heissler and Sellers 2014; Lowey and Risby 1971; Trybus 1994). Additionally, the binding of actin also induces enhanced ATPase activity in most myosins as described above. In other words, essentially no myosin acts alone within the cellular context, and the binding of additional cellular components is inextricably linked to myosin-dependent activity and function. Despite binding partner activation being hardly “unconventional,” several recent advances illuminate new mechanisms of activation and regulation.

Purification of myosin-7a typically results in a monomeric protein; however, a yeast-two-hybrid screen identified a binding partner (M7BP) which enhanced the motile properties of the motor (Liu et al. 2021). Interestingly, the stoichiometry of the motor to M7BP was measured at 2:2, resulting in oligomerization of the motor into a motile complex. The binding of M7BP to the motor relieves a head-to-tail auto-inhibition in Myo7a and tethers the complex to actin (Fig. 2). However, unlike the Myo5a complex, the M7BP-Myo7a complex can reorganize the actin into aligned bundles of filopodia-like protrusions (Liu et al. 2021). Thus, M7BP potentially represents a novel “all-in-one” type of activator, leading to simultaneous structural rearrangement, motility, and assembly of the correct actin structure for Myo7a motility. This activation is likely also present in Myo7b and may initiate the formation of the intermicrovillar adhesion complex through Harmonin-a (USH1C), and Ankyrin Repeat Protein, ANKS4B (Weck et al. 2016).

In addition to the above Myo7a studies, identification of binding partners for class-3 myosins (Myo3a/Myo3b in humans) has brought into focus a potential new theme seen across multiple classes: the activation of myosins by actin regulatory proteins. Espin1, an actin bundler associated with the polarity-aligned actin bundles microvilli and stereocilia, was found to release auto-inhibition of Myo3a and Myo3b (Liu et al. 2016). Two manuscripts published near concurrently also identified MORN4 as a Myo3a binding partner, which may provide lipid membrane association within stereocilia or microvilli (Gunther et al. 2020; Li et al. 2019). The Epsin1 finding adds to the growing list of myosin activators, which not only bind but also alter the cytoskeleton’s organization of the actin cytoskeleton. One surprising element of the Espin1-Myo3 binding is that Epsin1’s actin crosslinking ability is enhanced by the binding of Myo3 (Liu et al. 2016), indicating that both proteins are activated by the interaction.

A reasonable question follows: Why would a processive myosin motor require activation by a protein that will severely diminish its ability to move along actin? The answer may lie in Myo3’s highly specific cellular localization at the tips of stereocilia, in addition to its unusual incorporation of a signaling N-terminal S/T kinase domain. This localization and cellular function would require the motor to transition between motile (i.e., moving up the actin bundle of stereocilia) and sessile (i.e., maintaining its position at the tips of stereocilia) states (Fig. 2). Theoretically, a motor such as Myo3 could maintain a persistent motile state by either stepping at the exact rate as polymerizing actin (treadmilling) or repeatedly stepping off the filament tip and rapidly rebinding. However, the lessons learned from the energy studies of the SRX state in muscle suggest that the lavish expenditure of energy in this way would be evolutionarily unfavorable (Stewart et al. 2010). The highly specific cycling between motile and sessile states dependent upon an actin-binding protein has also been proposed for Myo1 (Tang and Ostap 2001). In microvilli, non-muscle tropomyosin prevents the binding of myosin-1 to actin (Fanning et al. 1994). In fact, several myosin classes are sensitive to the presence and isoform identity of the actin-bound tropomyosin (Clayton et al. 2015). Cytoskeletal tropomyosin isoforms are sorted and distributed to distinct actin structures within non-muscle cells (Manstein et al. 2020). Thus, Myo3 may follow a similar mechanism as proposed for Myo1, where newly polymerized bare actin filaments at the tips of sterocillia, or microvilli are available for binding (Tang and Ostap 2001). Yet, over time, the recruitment of Epsin1 or tropomyosin to the actin prevents the binding of specific myosin classes from defined regions of the actin filaments.

The only other motorized signaling molecules in humans, the class-9 myosins (Myo9a and Myo9b), may provide some additional insight (Bähler 2000; Sirotkin et al. 2000). Myosin 9b localizes to the tips of filopodia where it acts as a RhoA-specific GTPase-Activating Protein (GAP) (Vollmer et al. 2026). The mechanism for Myo9’s tip localization has been linked to crosslinking actin through the motor’s atypical loop-2 extension, which acts as a calmodulin-dependent actin tether (Liao et al. 2010; Saczko-Brack et al. 2016). The combined findings in Myo3 and Myo9 seem to suggest that actin crosslinking is required for localizing their signaling domains to the tips of actin-based protrusions (Houdusse and Titus 2021). This mechanism can occur either directly, as in Myo9, or through a regulatory binding partner, as in Myo3. During the writing of this review, a conserved 4-helix bundle required for filopodia-tip localization was identified in the tail of class-9 myosin, presenting the possibility of required regulatory binding partners like those seen in Myo3 (Vollmer et al. 2026). The full mechanism by which Myo3 and Myo9 are anchored to the tips of stereocilia and filopodia, respectively, will require further examination to clarify these potential models.

### Co-assembly of mixed myosins

Lipid membranes and intracellular cargos such as secretory granules, mitochondria, and Golgi are associated to multiple classes of motors including both microtubule and actin-based motors (Heaslip et al. 2014; Vale and Milligan 2000). A major advance over the past decade has been the discovery of multi-motor protein complexes comprised of different myosin isoforms that function independently of lipid structures. A breakthrough discovery was the identification of mixed bipolar filaments containing non-muscle myosin 2 (NM-Myo2) and myosin 18A (Myo18A) (Billington et al. 2015). This finding followed the characterization that all three human NM-Myo2 paralogs (NM-Myo2A/2B/2C) form mixed (heterotypic) filaments (Beach et al. 2014; Shutova et al. 2014). The incorporation of potentially 4 different myosins into a single bipolar complex allows for a novel level of biophysical and biochemical regulation. Multiple lines of evidence suggest that both NM-Myo2A and NM-Myo2B can act as pioneer molecules to assemble new bipolar filaments, then subsequently recruit the additional myosin paralogs (Shutova et al. 2017; Weißenbruch et al. 2022). The full assortment of regulatory systems that modulate the formation of NM-Myo2 bipolar filaments has been recently reviewed in depth elsewhere (Chinthalapudi and Heissler 2024; Quintanilla et al. 2023; Tidei et al. 2025).

Remarkably, the ratio of NM-Myo2B to NM-Myo2A increases over time in stress fibers. Within 10 h after formation, stress fibers initially formed through NM-Myo2A biogenesis become predominantly composed of NM-Myo2B (Shutova et al. 2017). This finding suggests that each NM-Myo2 paralog possesses a unique association or dissociation rate within a single bipolar filament. The regulation of the respective ratios of NM-Myo2 paralogs within a bipolar filament is believed to be related to the rate of dissociation for each paralog, not the association rate (Shutova et al. 2017). This is because the dissociation rate is dependent on the C-terminal tail, which is divergent in each paralog, leading to unique rates (Shutova et al. 2014). The C-terminal region of each paralog is subject to kinase and binding partner activity, providing a potential mechanism for differential regulation. The result is a tunable mechanical system where each actin structure can potentially recruit a singular combination of NM-2 paralog contractile units (Shutova et al. 2014).

Myo18A and Myo18B are examples of NM-2 binding partners which both require the C-terminal coiled-coil tail region for association to the NM-Myo2 bipolar filament (Jiu et al. 2019; Billington et al. 2015) (Fig. 2). However, recent evidence suggests that targeting of Myo18 also involves an N-terminal extension (Alexander et al. 2021). Myo18 motors do not self-assemble into large bipolar filaments, nor do they have any measurable motor activity (Guzik-Lendrum et al. 2013). Both Myo18A/B are instead believed to bind to the active and assembled NM-Myo2 bipolar filament to serve as a regulatory element within the complex (Jiu et al. 2019; Billington et al. 2015). One element of regulation that Myo18B coordinates is the stacking of 2 or more fully formed NM-Myo2 bipolar complexes (Jiu et al. 2019). This may be accomplished by creating a more stable binding platform for the addition of NM-Myo2 molecules than NM-Myo2 itself, as the turnover rate of Myo18 is much slower than that of NM-Myo2 paralogs within a bipolar filament (Jiu et al. 2019).

Myo18 gene products consist of several alternative splice isoforms. These isoforms enable an additional level of regulation to NM-Myo2/Myo18 complexes through the ability to bind isoform-specific binding partners. In Purkinje neurons, the Myo18A alpha isoform (Myo18Aα) is targeted to dendritic spines where it co-assembles with NM-Myo2 and directly binds to the RAC1/CDC42 guanine nucleotide exchange factor (GEF) β-PIX (Alexander et al. 2021). The GEF subsequently participates in a local signaling environment, including the assembly of actin and NM-Myo2 filaments, creating an apparent positive feedback loop for the maturation of spines (Alexander et al. 2021). Interestingly, the same biotin-ligase screen that identified Myo18B as a potential stress-fiber regulator in U2OS cells also identified Myo1c and MHY11 (smooth muscle myosin) (Jiu et al. 2019). These potential interactions have yet to be validated.

### Actin nanoscale organization: actin density, polarity, and architecture as a myosin regulatory mechanism

A recent push has unfolded to determine directly how the dynamic nanoscale order of actin acts as an independent regulatory mechanism for myosins. Nanoscale organization discussed here includes (1) filament density, (2) polarity, and (3) architecture, but is not limited to these topics (e.g., bending, twisting, tension, nucleotide state, post-translational modifications, etc.). These three components can be independent or correlated. For example, an Arp2/3 mediated complex could act with nucleation promoting factors in the formation of additional filaments (density). The new filaments would branch off existing filaments (architecture), with the plus-end of each new filament elongating at a 70° angle to the original filament (polarity) (Svitkina and Borisy 1999). At the core of the current understanding is the conclusion that the actin structures in cells are anything but uniform, and myosins have co-evolved to recognize the spatial segregation of this inhomogeneity (Santos et al. 2020).

Filament density’s effect on myosin activity can be directly measured through the investigation of motility along single vs. multifilament actin systems in single-molecule in vitro studies. At the single-molecule level, an individual non-muscle myosin’s interaction with a single actin filament follows relatively simple probabilistic rules (Rief et al. 2000). If the density is increased by one additional filament, the system follows the probabilistic rules, though now one must account for how the two filaments are ordered with respect to polarity and architecture (Rief et al. 2000; Ross et al. 2008). However, when the same motors are organized into multi-motor teams of 3 or more motors, which are geometrically free to probe the available actin binding landscape (e.g., embedded in a fluid-like lipid vesicle), unexpected emergent behaviors arise. These include increased velocities beyond that of this single motor (Nelson et al. 2014), altered filament switching (Lombardo et al. 2017), and motors transitioning between stationary, diffusive-like, and motile states (McIntosh et al. 2018; Walcott and Warshaw 2020). These emergent behaviors are also observed in cells (Heaslip et al. 2014) and are only understood once accounting for nanoscale organization as a regulator of multi-motor myosin complexes.

When a team of Myo5a motors bound to fluid-like lipid vesicles is challenged to navigate a 3D network of randomly polarized single actin filaments, two-thirds of vesicles fail to move in a directed manner (Lombardo et al. 2019). Instead, most vesicles are stationary or sub-diffusive. This behavior arises as single motors become strongly bound to actin filaments where the polarity faces in opposing directions. The resulting production of force from these motors produces an equal and opposite resistive load on the motors bound to oppositely polarized filaments (see Force Sensitivity) (Lombardo et al. 2019). For many classes of non-muscle myosins, the applied resistive loads result in the slowing of the ADP release and the capture of the motor in a state strongly bound to actin, effectively stopping motility (Fig. 2). Motility can only continue once either the actin filaments’ architecture changes (De La Cruz and Gardel 2015; Heaslip et al. 2014), the lipid cargo deforms (McIntosh et al. 2018), or some or all the myosin motors detach from the actin (Walcott and Warshaw 2020). Changing only the polarity, or both the architecture and polarity (by nucleation or branched filaments through ARP 2/3) of the actin filaments from randomly organized to be a mere 10% more polarity aligned, results in a massive regulatory shift. When slightly more polarity aligned, more than 50% of Myo5a-bound vesicles move in a directed manner (Lombardo et al. 2019). Remarkably, the paralogue Myo5c is only capable of processive motility along polarity-aligned actin bundles indicating the motor’s ability to be highly selective for actin’s nanoscale architecture (Sladewski et al. 2016).

Density of actin is a strong determinant of motility in actin networks. Specifically, a dense network of mixed polarity filaments can be a nearly impenetrable barrier to myosin-based motility (Heaslip et al. 2014; Miki et al. 2024). On the other hand, the polarity-aligned actin bundles of microvilli are evolutionarily optimized for the transport of nutrients across the gut and placental epithelia (Lombardo et al. 2024; Morales et al. 2023). In polarity-aligned networks, the influence of the density of actin regulators is minimized, where a few aligned filaments are nearly as effective as many aligned filaments. For example, consider that myosin-driven transport would be similar along a filopodium which has 15 polarity-aligned actin filaments compared to a filopodium with 20 polarity-aligned actin filaments. However, filament architecture within these aligned networks remains important. For instance, the presence of short polarity-aligned actin filaments positioned adjacent to an area of relatively longer, but similarly polarized filaments can create distinct microdomains of unique myosin activity in stereocilia (Liao et al. 2025).

What is the mechanistic basis by which nanoscale organization of actin regulates the properties of multi-motor teams or complexes? Biophysical and in silico models have shown that individual motors within any multi-motor complex with an elastic component are mechanically coupled. Thus, when actin filament polarity is aligned, motors at the leading edge of any complex experience a resistive load (delaying ADP release and detachment from actin) from the trailing motors, while trailing motors experience an assistive load (accelerating their ADP release and detachment from actin). This leads to preferential detachment from the trailing motors with a net result of increased velocity and reduced ATP usage per µm traveled by the motors (Nelson et al. 2014). When the actin filament’s polarity is not aligned, the forces from the motors are resistive, leading to an anchoring effect where little motion occurs, but still with reduced ATP usage. Thus, myosin-bound cellular cargos may use less energy than predicted from single-molecule in vitro studies, regardless of whether they are moving or stationary. These findings suggest the remarkable possibility that myosin motors may be evolutionarily optimized to detect actin density, polarity, and architecture for the purpose of energy efficiency. However, this theoretical conclusion has not yet been directly tested.

## Concluding comments

The work reviewed here has broadened our understanding of non-muscle myosin regulation, elucidating regulatory systems that extend beyond canonical biochemical switches. Rather, non-muscle myosins are regulated through intricate and often context-specific mechanisms that integrate the identity of associated lipids, binding partners, mixed-motor assembly, and the nanoscale organization of actin itself. Future models will require the unification of these emergent regulatory principles into predictive models that explain how diverse myosins collectively drive cellular behavior. For these models to advance the field, several outstanding questions must be resolved. For example, how are the binding interfaces between non-muscle myosins and lipids mediated at the atomic-structural level? Are these lipid-to-myosin interactions altered by the influences of additional protein-binding partners or the nanoscale organization of the actin to which the motor domain is bound? Complementing cellular data, single-molecule biophysical assays, atomic-resolution structures, and experimental or in silico molecular-dynamics information will be required to fully answer these questions. The combination of these approaches into wholistic understanding is beginning to emerge in the muscle myosin field, and extension into non-muscle myosin is an emerging area with vast potential.

## Data Availability

No datasets were generated or analyzed during the current study.
